# Canadian Occupational Performance Measure Supported by Talking Mats: An Evaluation of the Clinical Utility

**DOI:** 10.1155/2019/9367315

**Published:** 2019-01-22

**Authors:** Vita Hagelskjær, Mette Krohn, Pia Susanne Christensen, Jeanette Reffstrup Christensen

**Affiliations:** ^1^VIA University College, Gl. Struervej 1, 7500 Holstebro, Denmark; ^2^Bytoften Living and Activity Center, Bytoften 73, 7400 Herning, Denmark; ^3^The Research Initiative for Activity Studies and Occupational Therapy, Research Unit for General Practice, Department of Public Health, University of Southern Denmark, JB Winsløwsvej 9A, 5000 Odense, Denmark

## Abstract

**Background:**

Some clients with cognitive and communicative impairments after a brain injury are unable to participate in the Canadian Occupational Performance Measure (COPM) without support. The study originates from an assumption that some of these clients are able to participate independently in the COPM interview by using a visual material.

**Aim:**

The aim was to investigate the clinical utility of COPM supported by Talking Mats (TM) for community-based clients with cognitive and communicative impairments.

**Methods:**

Six clients (51–60 years) were included. After matching the visual material of TM to COPM, the COPM interview was administered twice with an interval of 10 days, once using TM and once without. Interviews were videotaped and studied by six evaluators.

**Results:**

The most obvious benefits of using TM as a supportive tool in the COPM interview were related to the first two steps of the COPM interview.

**Conclusion:**

Using TM in the COPM interview with clients with cognitive and communicative impairments after a brain injury is recommended as the basis for goal setting. The present study demonstrates a possibility to include a COPM interview to clients who had not been able to complete a COPM interview and thus start a rehabilitation process in a client-centered manner.

## 1. Introduction

Bytoften Living and Activity Center (BLAC) is a community-based rehabilitation center in Jutland, Denmark. Clients with impairments after a brain injury live and work there. To empower clients at BLAC in their rehabilitation process and to provide for their active participation in goal setting, the occupational therapists (OTs) use the Canadian Occupational Performance Measure (COPM). But the OTs meet challenges with clients who have severe cognitive and communicative impairments. Several studies show that using the COPM with these clients is associated with challenges due to difficulties in expressing their views, needs and wishes, and difficulties with comprehension, as well as with memory, overview, and insight; thus, they have difficulties in reporting their occupational performance (OP) [1–6].

In a client-centered approach and in promoting clients' active participation and empowering them in the rehabilitation process, it is important that clients participate in formulating their own goals [7–11]. Several studies show that active involvement in goal setting promotes clients' motivation, increases empowerment and participation in therapy, and correlates with treatment outcomes [11–13]. The COPM suggests that caregivers or relatives take the role of respondents for clients who are not eligible for COPM due to cognitive impairments [14]. Murphy and Cameron draw attention to the fact that “… the boundary between giving support and making decisions for people with intellectual disability is blurred and that relatives and caregivers often overstep into the latter … particularly if the individual has a communication impairment” [15]. Further, goal setting based on relatives' or caregivers' perspectives of OP “does not fit comfortably with providing a client-centered approach” [16]. At BLAC, the OTs involve caregivers or relatives in the COPM as suggested. But some of these clients might be able to participate independently in the COPM, despite their cognitive and communicative impairments, if an alternative communication method supported the COPM thus enhancing the dialogue between clients and OTs.

Talking Mats (TM) is a low-technology communication tool [17]. It is described as a strategy to help people with communicative impairments express their opinions [18–20]. It consists of a mat and a set of pictograms including topics, a visual scale, and options. During the dialogue, pictograms are placed on the mat by the client indicating the topic of the conversation. TM allows open questions and empowers the clients to express their thoughts about different topics related to everyday activities [19]. Studies investigating the effectiveness of TM in relation to communication, shared decision-making, and goal setting have shown potential with different client groups, including people with dementia, Huntington's disease, aphasia, and intellectual disabilities [15, 17, 18, 21, 22].

Thus, the primary aim of this study was to investigate the clinical utility of COPM supported by TM, for clients with cognitive and communicative impairments after a brain injury, in order to promote empowerment and a client-centered approach. The secondary aims were to evaluate to which extent the steps of the COPM were completed and how the use of TM affected the quality of the dialogue in the COPM interviews.

## 2. Materials and Methods

**Figure 1:**
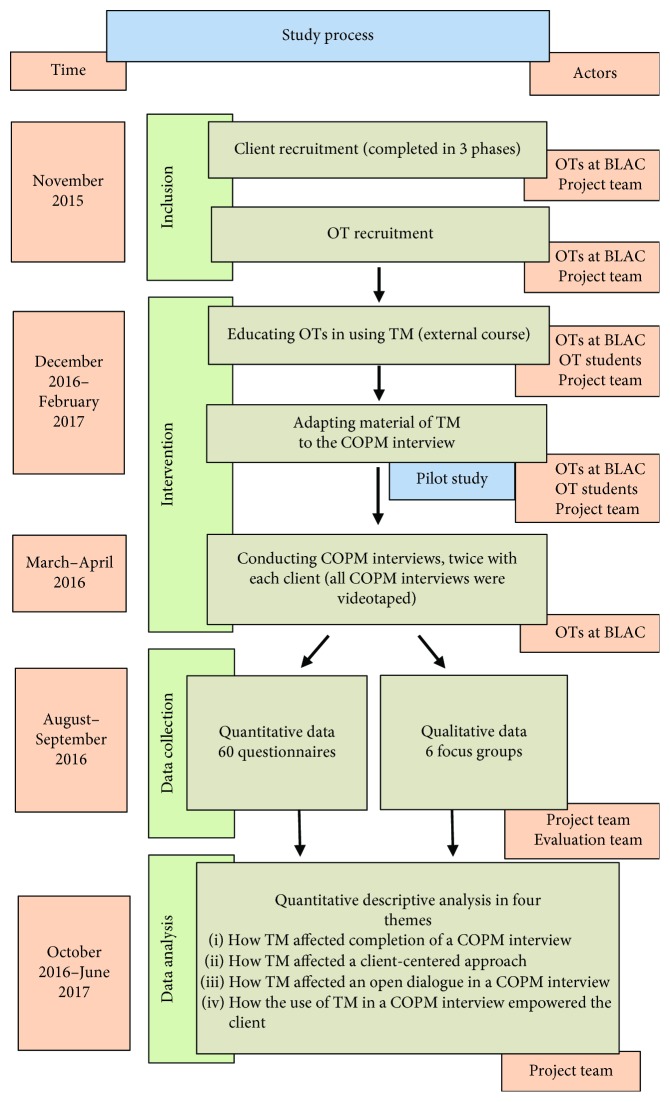
The study process.

The study was designed as a process evaluation with a focus on application in practice where the involved OTs played an important role in all steps of the evaluation. All clients included gave their informed and written consent, and the study was approved by the Data Protection Agency. The project team planned and completed the evaluation, which was conducted by the first three authors. The evaluation team assessed the data in relation to the aims of the study, which consisted of the project team, the OTs who conducted the interviews, and a manager at BLAC, all with experience in assessing video materials.  Figure 1 illustrates the study process.

### 2.1. Inclusion

**Figure 2:**
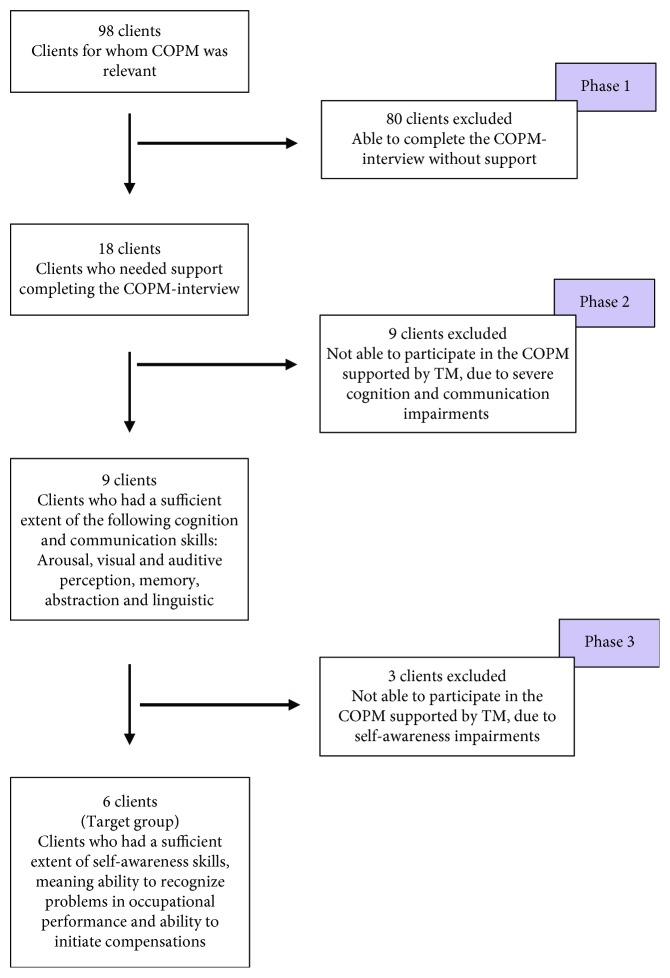
Client recruitment.

Client recruitment was based on the OTs' insight into the client's competences and was completed in three phases (Figure 2). At BLAC, the COPM interview was used as a basis for goal setting for all clients if possible. When the study began, 98 clients were admitted to BLAC.

#### 2.1.1. Client Recruitment Phase 1

In phase 1, 80 clients were excluded from the study because they were able to complete the COPM interview without any support. This left 18 clients who were not able due to doubts about the quality of the completed COPM. Either the COPM did not support the goal setting process or the clients had difficulties expressing their perspectives and doubts regarding whether the expressions were the client's, the relative's, or the OT's, and there were doubts whether the clients understood the questions asked.

#### 2.1.2. Client Recruitment Phase 2

In phase 2, nine clients were excluded due to severe cognitive and communicative impairments, leaving nine clients with sufficient cognitive and communication skills (Figure 2). These criteria were chosen based on the World Health Organization International Classification of Functioning, Disability and Health [23], and they were inspired by evaluations of the effectiveness of TM used in clients suffering from dementia, Huntington's disease, aphasia, and intellectual disabilities [15, 18, 21, 22, 24, 25].

#### 2.1.3. Client Recruitment Phase 3

In phase 3, three clients were excluded due to their self-awareness impairments. Thus, six clients had sufficient skills of self-awareness, which refers to the ability to recognize problems in OP and the ability to initiate compensations. The clients' level of awareness was based on the hierarchical model of awareness [26], and the inclusion of a partial emergent awareness was required.

#### 2.1.4. OT Recruitment

Fourteen OTs were employed at BLAC at the beginning of the present study. To be included in the study, the OTs needed to have familiarity with the COPM, a willingness to take part in a one-day course in the use of TM, and knowledge of the recruited clients' overall situation, and they must have completed at least one COPM with the use of TM as a supportive tool. These requirements included three OTs.

### 2.2. Intervention

The COPM was conducted twice with each client, once using TM as a supportive tool and once without. The interviewer was aware to carry out both COPM interviews as objectively as possible, but no further strategies were used to counterbalance the two interviews.

#### 2.2.1. The COPM Interview

The COPM is a client-centered outcome measure of a client's self-perception of his or her OP issues and provides the basis for goal setting [14]. The COPM is administered as a semistructured interview in five steps. In step 1, the OT interviews the client about his or her OP within three areas of occupation: self-care, productivity, and leisure. In step 2, the client rates the importance of the identified OP issues on a 10-point scale. In steps 3 and 4, the client chooses up to five issues that seem most important or pressing. For each of the chosen issues, the client scores performance and satisfaction on 10-point scales [14]. Step 5 is reassessment, which is not a part of this study.

#### 2.2.2. Talking Mats

The TM framework is a low-technology communication method which helps clients with communication impairments to understand and respond more effectively [15]. It offers mats and three sets of picture symbols: topics, options, and a visual scale (see an example in Appendix 2). The topic could be pictures symbolizing activities, the environment, relationships, or self-care. The options relate to the topic, e.g., listening to music or visiting friends. The visual scale allows the clients to indicate their feelings about each topic, such as happy or unsure. It is an important feature of TM in which the interviewer asks open questions to ensure that the interviewers' perspective does not influence the client. The clients are encouraged to comment on both positive and negative feelings and to look back over their mats in order to alter or confirm their views. The interviewer can use as many mats as appropriate for each client [17].

#### 2.2.3. Educating OTs in Using TM

The first step in preparing the intervention was completion of a one-day external course in the use of TM. The course was delivered by an accredited Talking Mats trainer from the “Kommunikationscentret” in Hillerod, Denmark. All OTs from BLAC, a group of OT students, and the members of the project team attended the course. The next step was the process of adapting an image material from TM to the COPM interview, as described in the following (see an example in Appendix 2 for mats completed with the steps of the COPM interview).

#### 2.2.4. Adapting Material of TM to the COPM Interview

This step included the development of image material and the guideline for the use of TM as a supportive tool in the COPM interview. A group of OTs from BLAC developed the image material under the supervision of the project team. The image material was developed to support the steps of the COPM but without altering the COPM method and included a set of pictograms related to the COPM OP issues (self-care, productivity, and leisure) and the COPM rating scales (importance, performance, and satisfaction). For example, the pictograms related to self-care included dressing and eating, pictograms related to productivity included cleaning and cooking, and pictograms related to leisure included visiting a restaurant and reading. In addition, the project team developed the guideline with recommendations before conducting a COPM interview when using TM as a supportive tool (Table 1). Two pilot interviews were completed, and adjustments to the guideline and image material were made to increase the internal validity.

#### 2.2.5. Conducting the COPM Interviews

The same OT conducted the COPM interview twice with each client, once using TM as a supportive tool and once without. The mean interval of 10 days was found between the two interviews, and all were videotaped. The video material was the basis for the following process of data analysis and the assessment of the quality of the COPM interviews.

### 2.3. Data Analysis

To illuminate the outcomes and possible reasons for failure or success when using TM in the COPM, both quantitative and qualitative data were analysed and assessed. These were analysed and assessed over the course of three days with participation of the evaluation team, where the evaluation team reviewed the videos of the COPM interviews with all six clients. After watching each videoed interview, the members of the evaluation team individually scored a questionnaire. After scoring both interviews on each client, the evaluation team participated in a focus group interview.

#### 2.3.1. The Questionnaire

The questionnaire (Appendix 1) covered three areas related to the level of client-centeredness:

- The client's role and perspective on how the interview affected his or her engagement, understanding, and reflection
- The OT's role, which refers to how the interview form affected the OT's understanding of the client's views and to what extent the COPM interview was adapted by the client
- To what extent the first four steps in the COPM interview were completed

The questionnaire consisted of both closed and open questions. The closed questions were answered on a 4-point Likert scale: 1: to a large extent; 2: to some extent; 3: to a lesser extent; 4: not at all; and 5: not applicable/do not know. In the open questions, the evaluators were asked to describe the signs they identified that were decisive for their scores, i.e., eye contact, body language, tone of voice or sounds, initiative, and reaching out for the pictograms. Altogether, 60 questionnaires were completed (five evaluators scored two interviews on each of the six clients).

#### 2.3.2. The Focus Group Interviews

The focus group interviews with the evaluation team were conducted immediately after the individual scoring of the questionnaire. The interviews concerned differences in the quality of the COPM interviews with and without the use of TM in relation to each client; see the interview guide in Table 2.

Both the quantitative data from the questionnaires and the qualitative focus group interviews were analysed to contextualize the results and to describe the utility of the COPM supported by TM as a supportive tool. The analytic strategy was inspired by Yin's recommendations [27]. Both the qualitative and quantitative data of each client were analysed separately, identifying the outcomes and main topics relevant for the study. The quantitative data were analysed descriptively with each client as one unit, according to the case study method [27]. Each interview was transcribed and analysed by dividing text into meaning units and labelled with codes. Afterwards, the codes of all six interviews were compared and sorted into categories. Discussion of the content of the categories resulted in four themes, all related to the quality of the COPM [28]. Finally, outcomes and themes were analysed across the six cases, using the qualitative analysis as supporting evidence for the quantitative data.

## 3. Results

The study included four men and two women (51–60 years). An overview of the clients' gender, age, cause of brain injury, and communication skills is presented in Table 3.

The results of how the two types of interview influenced the quality of the COPM are presented within four themes: (1) how TM affected the completion of the COPM interview; (2) how TM affected a client-centered approach; (3) how TM affected the quality of the dialogue in the COPM interview; and (4) how the use of TM in the COPM interview empowered the client.

### 3.1. How TM Affected Completion of the COPM Interview

Table 4 shows to what extent the steps of the COPM were completed, comparing the COPM interview supported by TM to the regular COPM interview.

It shows that the benefits of using TM as a supportive tool were related to the first two steps of the COPM, which identified the client's perception of the OP and the importance ratings of the daily activities. In a total of 30 scores, 25 was scored to complete steps 1 and 2 to a higher degree, when using TM, compared to a score of 16 in the regular COPM interview. Studying the videoed interviews, it was obvious that especially two of the clients had the advantage of using TM during the first two steps of the COPM. An evaluator said, concerning these two clients, “The OT does not have to guess what they [client 2 and 5] mean, she [the OT] can get clear answers, and you actually feel that their [OT and clients] dialogue goes in depth …” With client 2, the benefit from using TM was obvious, as the client was unable to identify OP issues in the regular COPM interview, whereas in the COPM interview using TM, the client identified 18 OP issues. With client 5, the descriptions of the OP were more nuanced when using TM. Comparing the scores concerning to what extent steps 3 and 4 were completed, the evaluation team concluded there was no obvious difference in the two types of interview. Altogether, the evaluation team agreed that five of the six clients profited from using TM in the COPM, while one did not. This will be elucidated in the following.

### 3.2. How TM Affected a Client-Centered Approach

The members of the evaluation team scored the items *understanding*, *reflection*, and *engagement* for each client in both types of interview. Since the item *understanding the question* is a condition for reflecting, the focus was on the items *reflection* and *engagement*. The scores of the clients' engagement and reflection in the two different types of COPM interviews are presented in Table 5.

The scores of the clients when using TM increased the engagement with clients 2-5. No differences in engagement were seen with clients 1 and 6. The evaluation team also noted that clients 2-5 supplied their answers with more elaboration and explanation in the COPM interview when using TM and that these interviews had more characteristics of a dialogue with interaction between the client and the OT. With client 2, the evaluation team even noted that this client had a considerably higher arousal, attention, and participation with the support of TM and that her perspectives were clearer.

Clients 1-5 showed increased ability to reflect on questions in the COPM with TM. These clients used the rating scales in a more diverse way, repositioned the TM images, and paused before answering. This was the opposite with client 6, who increased his reflections in the regular COPM compared to the COPM supported by TM, as he explained and expressed more perspectives. Overall, he seemed to be at more ease when talking about his daily activities in the regular COPM compared to the COPM with TM.

### 3.3. How TM Affected the Quality of the Dialogue in the COPM Interview

In four of the six cases, the use of TM increased the quality of the COPM interview because of a more successful dialogue. In these four cases, there was a match between the client's assumptions and the TM material, and thus, an in-depth dialogue, an increased client reflection, and the OT's understanding of the client's perspectives were supported. In the last two cases, TM did not influence the COPM interview positively, as the dialogue became too closed and structured.

TM as a supportive tool in the COPM interview primarily affected the first two steps of the COPM, as seen in Table 4. The evaluation team noted within step 1 that the visual material in TM promoted the client's achievement and kept an overview of the dialogue. An evaluator said, “His overview decreases in the [regular] COPM when he is supposed to prioritize the most important occupational issues. On the other hand, he is actually able to do so, when TM is involved …” TM made it clear to both clients and OTs what topic the conversation was concerned with and that TM motivated most clients. TM provided an overview of the client's answers after each step of the COPM. The evaluation team also found that TM qualified the preparation of the COPM. An OT said, “… having made the decision of using TM as a supportive tool, the process of prioritizing symbols and images, designing rating scales and considering how to use the mats and images, surely qualified my preparation ….”

Using TM also promoted clearer answers from the clients. Because of the images and mats, the OTs could not continue the COPM to the next step until the current step was completed. An evaluator said, “… the symbol must be physically placed on the mat, before the interview continues ….” The evaluation team noted that the use of TM decreased doubts and the need for clarifying questions. The visual material made answers clearer and gave the clients the opportunity to ask questions that were not possible without the visual material (see example of mat and pictures in Appendix 2); i.e., using TM promoted the possibility of asking open-ended questions for client 4, whereas the regular COPM interview was dominated by closed questions. Client 6 was the only client who expressed that using TM affected the quality of the dialogue in a negative way. An evaluator said, “... but I do not think he gets the same opportunity in COPM with TM to expand his answers … I have this feeling that he would have liked to say something more ....”

### 3.4. How the Use of TM in the COPM Interview Empowered the Client

The evaluation team noticed examples of clients taking the lead in the COPM, being able to follow the progress, and maintaining an overview to a higher degree than normal when using TM. An evaluator said the following about client 5: “… in the [regular] COPM, it is much more the OT who controls the course of the battle and he [the client] has no opportunity to play an active role and be in control of the situation and decide what to talk about. TM simply promotes this….” It was clear that TM promoted clients' empowerment when they were able to point insistently at the pictures, used images and mats to make themselves understandable, and came forward with their opinions. Furthermore, it appeared that client 5 focused on the images and mats rather than on the OT and her direct recognition of his responses. The evaluation team found that client 5's answers were more contemplated, concise, and uncertain in the regular COPM compared to the COPM supported by TM. Thus, the interviewer effect seemed to be reduced when using TM as a supportive tool. The same was identified with client 1: “... in the COPM, the OT sits on her side of the table with the paper, and she take notes and takes some time to do that. Here [in COPM with TM] they are always in a position to put things up and he [the client] can constantly follow the process. In some sense, he has more ownership in COPM with TM. He [the client] takes responsibility for the mat and begins to reorganize the images, and asks for some images to be taken off again. It shows that he [the client] has more influence on the situation and a larger overview than within the regular COPM.” With client 4, the OT spoke more in the regular COPM than when using the TM. Client 4's influence was more applicable in COPM with TM: “You [OT] speak much more during the regular COPM-interview, trying to get her [client 4] opinion forward. In COPM with TM, you speak less, and she [client 4] has much more sound in her voice, supporting what she thinks and she takes the initiative to take the images. In fact, she becomes a bit impatient at one point [in the regular COPM-interview] ….”

## 4. Discussion

The main findings showed that using TM increased the chances for clients with cognitive and communicative impairments following a brain injury to fill out the COPM, especially within the two first steps. In most cases, TM empowered and increased a client-centered approach and affected the quality of the dialogue between the clients and the OTs in a positive way.

### 4.1. How TM Affected the Completion of the COPM Interview

The benefits of using TM as a supportive tool in the COPM interview seemed to be related to the first two steps of COPM. This might be due to the OT's lack of routine in using TM as a supportive tool when choosing and applying an appropriate number of mats [29]. Another explanation could be that the interviews supported by TM took a longer time than the ordinary COPM interview (additional 10–50 min). Consequently, the client might have spent more resources in completing the first half of the interview with TM than the second half due to fatigue or loss of concentration. Thus, dividing the interview into two parts might have been beneficial. This is in line with recommendations by Law et al., who point out that the OT should not hesitate to be creative and to complete the COPM interview in an adaptive way [14]. Another perspective could be that TM is new to the client, and more experience with TM may change the result. The *Evaluation of the Talking Mats Method—Used with People with Dementia* states that people with dementia found it easier to use TM on the third attempt [30]. A last consideration could be that TM is simply more beneficial in the first two steps in the COPM interview in order to provide structure and overview in the beginning for the client.

### 4.2. How the Use of TM Empowered the Clients and Affected a Client-Centered Approach

The study shows that the use of TM as a supportive tool in the COPM interview has the potential to increase the clients' empowerment by supporting their ability to keep an overview, actively participate by placing and replacing images, and constantly follow the progress of the interview. These findings are in agreement with results from other studies related to dementia [20, 24, 31]. The evaluation team found it obvious that what really mattered in empowering the client was how the OT played the role as an interviewer and how the interview form was prepared. According to Brinkmann and Kvale, a semistructured and qualitative interview always has an inherent asymmetry between two parties, as it is the interviewer who asks, initiates, and defines the interview [32]. The interviewer in the COPM interview strives for a free and equal dialogue, and if the interviewer is not aware of the asymmetric relationship, the interview situation can become a manipulative conversation with a more or less hidden agenda, with the right of interpretation by the interviewer [32]. When the interviewed has cognitive and communicative difficulties, enabling the client's power is a challenge for the OT. Thus, the role of the OT as an interviewer should be given extra attention. Therefore, the interviewer should thoroughly assess whether his or her skills meet the requirement of the following issues at the same time. The interviewing skills, including the awareness of potential asymmetry, manage to adapt the interview form to the client's abilities to participate and keep an overview, succeed in engaging the client in the interview and in giving the client ownership, and ensure that the client is being heard and understood. Being aware of the interviewers' influence when interviewing people with little or no speech and the risk of “putting words into the interviewee's mouth,” when selecting the themes before the interview, is also mentioned by Brewster [33]. Even though the OT preselected images that were considered to be relevant to the client when using TM in the COPM interview in this study, the evaluation team identified an example (client 6), where “words seem to be put in the client's mouth”: “[...] in COPM with TM, it is the OT who is in power of controlling the direction of the interview. In the regular COPM, it is rather him [client 6] who starts and you [the OT] as interviewer, who guides him to move on from the path that he himself initially chose. In COPM with TM, the OT puts out the tracks and he will follow them. I think, seeing this, there is a risk that what is really important to him, does not appear.” Based on this interpretation, the evaluation team concluded that TM was not used as intended with this client.

### 4.3. The Utility of TM and How TM Affected the Quality of the Dialogue in the COPM Interview

Whether or not TM is relevant should be based on the OT's knowledge of the clients' cognitive and communicative capacity, and we suggest that OTs follow our inclusion criteria. If the client has a higher cognitive level of function or communicative skills than the participants in this study, TM might restrict the dialogue and not unleash the full potential of the client. Conversely, if the client does not have the sufficient cognitive level of functioning to understand and engage with the remedies used in TM, or if visual impairment prevents the client from seeing symbols and mats, TM involvement could adversely affect the client's motivation and commitment in a negative way. Despite the use of our inclusion criteria, we experienced that TM involvement was inhibiting the quality of the interview in a negative way for one of the clients. This client wished to say more and to elaborate on the subjects more than the interview form enabled.

It is also relevant to consider to which extent the OT's experience in using TM with COPM can impact the quality of the dialogue. Schön's description of “The Reflective Practitioner” [29] points out that the more familiar and experienced a practitioner is with a particular method, the more the practitioner will be aware of the signs and signals during the interview and able to immediately graduate and adapt accordingly to “reflection in action” [29]. This point of view is supported by the COPM manual: “the therapist should follow the client's lead in dealing with different areas as they arise in the interview” [14]. Based on these considerations, it might be relevant to repeat the study with OTs who are more experienced in the application of TM to the COPM.

### 4.4. Methodological Considerations

The study had several limitations. Firstly, the small number of clients included provided limited evidence for the results. Secondly, the study only took the perspective of professionals, which may seem inappropriate, since the study was interested in clients' active participation. Thirdly, the design of the study could be questioned. Comparing two methods of completing the COPM with the same client 10 days apart may have affected the validity. In addition, the OTs were relatively inexperienced in using TM. A larger-scale study and a study including OTs with more experience using TM as a supportive tool in the COPM interview would increase the validity. Still, because of the complexity of these clients' situations and with their different reasons for not being able to complete the regular COPM interview, the design is considered appropriate. In spite of the limitations, the study could contribute to OTs' praxis, which focuses on adults with communication and cognition impairments following a brain injury.

In the present study, TM supported the steps and the completion of the COPM interview, as the aim was to fill out the COPM. The COPM was the focus at all times, and the method was not altered in any way. TM was used instead of help from relatives or caregivers to find an alternative, which may have been a better way to fill out the COPM and thereby enhance a client-centered practice.

## 5. Conclusion

It seems that TM has the potential to support a client-centered approach, empower the clients, and increase the quality of the dialogue, when used as a supportive tool in the COPM. With this study, we therefore carefully suggest using TM as a supportive tool in the COPM for certain clients with cognitive and communicative impairments following a brain injury, when COPM is used as a basis for goal setting rather than an outcome-measuring tool. Our findings confirm the results of other studies, in that OTs should be careful when using COPM as an outcome measure with clients with cognition impairments. Finally, we recommend that a larger-scale study should be conducted in order to confirm our results before a final recommendation to use TM in the COPM is given.

## Figures and Tables

**Table 1:** The guideline—overview of content.

The guideline supported preparation of the COPM interview using TM as a supportive tool. The purpose of the guideline was to support promotion in the client-centered approach during the COPM interview. It followed the five steps of the COPM interview. (i) Consider mind mapping the activities, interests, and roles of the client (ii) Do you need information from relatives, caregivers, or others? (iii) Which structure of the interview is preferable? (completing the interview and rating one OP issue at a time or finishing the interview and completing the rating scales afterwards) (iv) To what extent do you expect the client to manage the pictures/symbols? (v) Consider the formulation of questions in full for the client concerned (vi) Choice of documentation, either the original COPM form or photos of the mats after each step COPM step 1: identifying occupational performance problems (i) Choice of images: size, amount, or type (ii) Choice of relevant division of the mat COPM step 2: rating importance of identified activities (i) Choice of mats and rating scales. 10-point scale is optimal. Alternatively, choose 10-point scales with images or words. Be aware of the validity COPM steps 3 and 4: scoring OP and satisfaction (i) The process of choosing the most important OP issues allows for reflection and validation of the clients' answers. Use the same mat used in step 2 (ii) Choice of rating scales. 10-point scale is optimal. Alternatively, choose scales with images or words. Be aware of the validity. Lowest rating option is placed on the left side of the mat COPM step 5: reassessment (i) Choose the same scales as in steps 3 and 4

**Table 2:** Interview guide, focus groups.

Research questions: how is the utility of COPM using TM as a supportive tool? Does it promote the client-centered approach? How, when, and under which circumstances? Consensus was not the purpose of the focus groups; rather, the purpose was to identify different perspectives. Scores of the individual questionnaires hung on the wall and were visible to everyone. The scored questionnaires were the basis for the interview. 1. Preliminary questions Started with a two-minute individual reflection, followed by a round where everyone got the chance to talk (a) *What are your immediate thoughts?* (b) *What is the first thing you notice?* 2. In-depth questions, following themes in the questionnaire Client role: (a) *How did the interview form affect the client's engagement, understanding, and reflection?* (b) *Did the use of TM make a difference in the client's role during the COPM interview?* (c) *How? Pros and cons?* (d) *Consider the possible reasons. Give examples (how, when, and under what circumstances)* OT role: (a) *How did the interview form affect the OT's understanding of the client's views?* (b) *Did the use of TM make a difference in the OT's role during the interview?* Aim of COPM: (a) *Did TM make a difference in reaching the goals of a COPM interview?* (b) *How? Pros and cons?* (c) *Consider the possible reasons. Give examples (how, when, and under what circumstances)* 3. Final questions (a) *Is there something we need to get around?* (b) *Are there other important experiences you have seen?* (c) *What is your immediate assessment of the effect of using TM in the COPM interview, considering this particular client?* (d) *How do you assess the client-centered approach, comparing the COPM interview with and without the use of TM?*

**Table 3:** Included clients.

Client	Age	Type of brain injury	Communication skills
(1) Male	55	Multiple sclerosis	Uses alphabet board, answers by eye blink
(2) Female	60	Cerebral palsy	Uses alphabet board and pictograms, verbally expresses “yes” and “no”
(3) Male	51	Trauma	Uses pictures and pictograms, answers by mimics and body language
(4) Female	55	Stroke	Uses touch pad, expresses herself by body language and sounds
(5) Male	52	Cerebral palsy	Expresses himself verbally
(6) Male	51	Stroke	Expresses “yes” and “no” by nodding and shaking his head

**Table 4:** To what extent the steps of the COPM were completed.

	COPM with TM			COPM		
	To a higher degree	To a lesser degree	Not scored	To a higher degree	To a lesser degree	Not scored
COPM step 1 (problem definition)	25	2	3	16	9	5
COPM step 2 (rating importance)						
COPM step 3 (scoring performance)	20	7	3	22	4	4
COPM step 4 (scoring satisfaction)	19	6	5	21	4	5

The table shows the distribution of the total scores. Each of the five evaluators scored six clients (*n* = 30). To a higher degree combined scores “to a large extent” and “to some extent.” To a lesser degree combined scores “to a lesser extent” and “not at all.”

**Table 5:** Clients' engagement and reflection in the two types of interviews.

			Clients' engagement						Clients' reflection					
			COPM with TM			COPM			COPM with TM			COPM		
Client	Age	Type of brain injury	To a higher degree	To a lesser degree	Not scored	To a higher degree	To a lesser degree	Not scored	To a higher degree	To a lesser degree	Not scored	To a higher degree	To a lesser degree	Not scored
(1) Male	55	Multiple sclerosis	4	1	0	4	1	0	3	2	0	2	3	0
(2) Female	60	Cerebral palsy	4	0	1	0	4	1	4	0	1	0	4	1
(3) Male	51	Trauma	4	1	0	1	4	0	3	1	1	0	3	2
(4) Female	55	Stroke	4	0	1	3	0	2	4	0	1	2	1	2
(5) Male	52	Cerebral palsy	5	0	0	0	4	1	4	1	0	0	4	1
(6) Male	51	Stroke	5	0	0	5	0	0	2	3	0	4	1	0

The table shows the distribution of the scores of the five members in the evaluation team (*n* = 5). To a higher degree combined the scores “to a large extent” and “to some extent.” To a lesser degree combined the scores “to a lesser extent” and “not at all.”

## Data Availability

The data that support the findings of this study are available from the corresponding author, JRC, upon reasonable request.

## Supplementary Materials

Supplementary Materials Appendix 1: “questionnaire covering three areas related to the level of client-centeredness” shows the questionnaire that the evaluation team has used to score the compliance and the quality of the COPM interview. Appendix 2: “example of a completed COPM interview supported by Talking Mats.” The example includes occupational performance within self-care, productivity, and leisure.
