# Supplementary material for: Canadian Occupational Performance Measure Supported by Talking Mats: An Evaluation of the Clinical Utility
Source: Occup Ther Int. 2019 Jan 22;2019:9367315. doi: 10.1155/2019/9367315 (PMC6362486; doi:10.1155/2019/9367315)

**Appendix 1: Questionnaire covering three areas related to the level of client-centeredness**

| **Name of client:**  **Name of evaluator:** | **□**COPM (1) **□**COPM (2) **□** COPM with Talking Mats (1) □ COPM with Talking Mats (2) |
| --- | --- |

**The role of the client**

| **1.** | **To a large extent** | **To some extent** | **To a lesser extent** | **Not at all** | **Not applicable/**  **don´t know** | **Observations and reflections** |
| --- | --- | --- | --- | --- | --- | --- |
| **The client is engaged in the interview** |  |  |  |  |  |  |
| Possible signs identified:  • Looking at or having eye contact with the occupational therapist  • Changes in voice, breathing, body language  • Receives or reach out for the pictures  • Looking at the pictures  • Place the pictures or indicate where the pictures are to be placed  • Select pictures | | | | | |

| **2.** | **To a large extent** | **To some extent** | **To a lesser extent** | **Not at all** | **Not applicable/**  **don´t know** | **Observations and reflections** |
| --- | --- | --- | --- | --- | --- | --- |
| **The client understands the questions** |  |  |  |  |  |  |
| Possible signs identified:  • Nuances in the answers  • Changes in voice, breathing, body language  • Smiles  • Receives or reach out for relevant pictures  • Looking at relevant pictures  • Place the pictures or indicate where the images are to be placed  • Selects relevant pictures | | | | | |

| **3.** | **To a large extent** | **To some extent** | **To a lesser extent** | **Not at all** | **Not applicable/**  **don´t know** | **Observations and reflections** |
| --- | --- | --- | --- | --- | --- | --- |
| **The client reflects on the questions** |  |  |  |  |  |  |
| Possible signs identified:  • Explains his or her answers  • Is being critically and/or reassigns the pictures in the review  • Consider the placement of the pictures | | | | | |

**The role of the occupational therapist**

| **4.** | **To a large extent** | **To some extent** | **To a lesser extent** | **Not at all** | **Not applicable/**  **don´t know** | **Observations and reflections** |
| --- | --- | --- | --- | --- | --- | --- |
| **The occupational therapist understands the clients' views** |  |  |  |  |  |  |
| Possible signs identified:  • The occupational therapists use of clarifying questions  • The occupational therapist interprets, guesses and/or exhibits doubts about the given answers | | | | | |

| **5.** | **To a large extent** | **To some extent** | **To a lesser extent** | **Not at all** | **Not applicable/**  **don´t know** | **Observations and reflections** |
| --- | --- | --- | --- | --- | --- | --- |
| **The occupational therapists approach to the interview is adapted to the client** |  |  |  |  |  |  |
| Possible signs identified:  • The scope and structure of the interview  • Questioning, e.g. closed, opened and or leading questions  • Size and clarity of the pictures or symbols  • Numbers and designs of the mats  • Selected scales are suitable for the client | | | | | |

**Fulfillment of goal in relation to COPM**

| **6.** | **To a large extent** | **To some extent** | **To a lesser extent** | **Not at all** | **Not applicable/**  **don´t know** | **Observations and reflections** |
| --- | --- | --- | --- | --- | --- | --- |
| **The clients' perception of own needs for daily activities is identified** |  |  |  |  |  |  |

| **7.** | **To a large extent** | **To some extent** | **To a lesser extent** | **Not at all** | **Not applicable/**  **don´t know** | **Observations and reflections** |
| --- | --- | --- | --- | --- | --- | --- |
| **The client assesses own occupational performance of identified daily activities** |  |  |  |  |  |  |

| **8.** | **To a large extent** | **To some extent** | **To a lesser extent** | **Not at all** | **Not applicable/**  **don´t know** | **Observations and reflections** |
| --- | --- | --- | --- | --- | --- | --- |
| **The client assesses his own satisfaction with the occupational performance of identified daily activities** |  |  |  |  |  |  |

**Other**

| **9.** |  |
| --- | --- |
| **Other observations and reflections** |  |

**To be answered after the 2nd interview**

| **10.** | **To a large extent** | **To some extent** | **To a lesser extent** | **Not at all** | **Not applicable/**  **don´t know** | **Observations and reflections** |
| --- | --- | --- | --- | --- | --- | --- |
| **The use of Talking Mats promotes the utility of the COPM-interview for the client, according to section. 6, 7 and 8** |  |  |  |  |  |  |

**Appendix 2. Example of a completed COPM-interview supported by Talking Mats**

**- The example includes occupational performance within self-care, productivity and leisure**

**Step 1: Problem definition.** Pictures placed by the negative smiley are the occupational performance problems identified by the client. Pictures placed by the positive smiley are not associated with problems of performance.


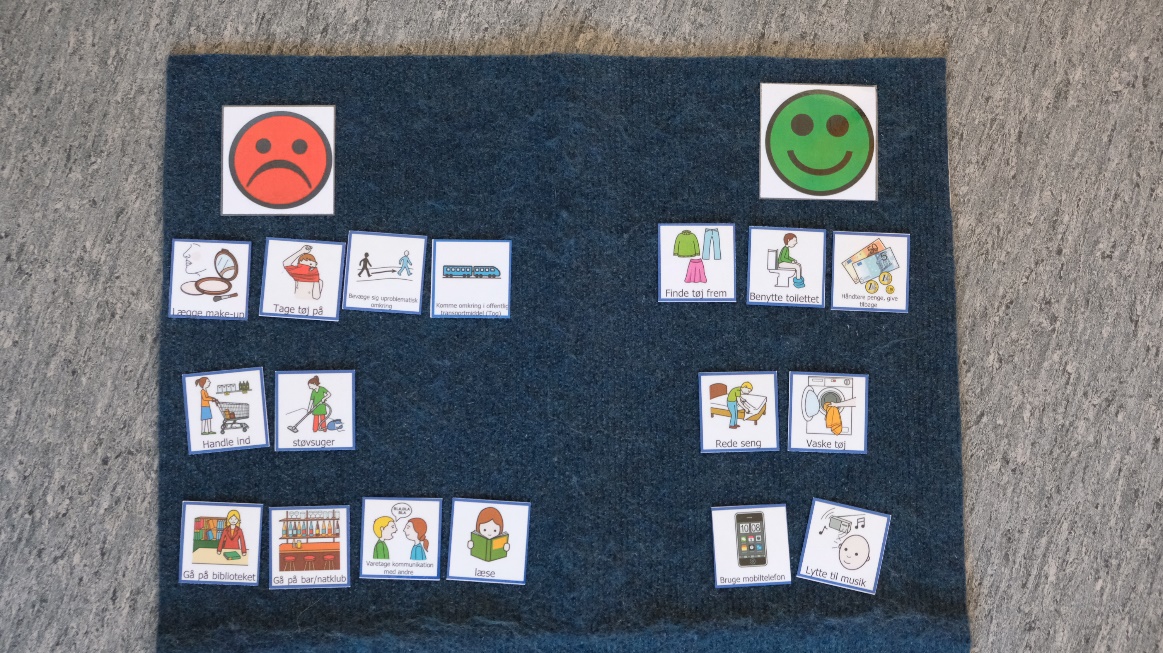


**Step 2: Rating importance of occupational performance problems**


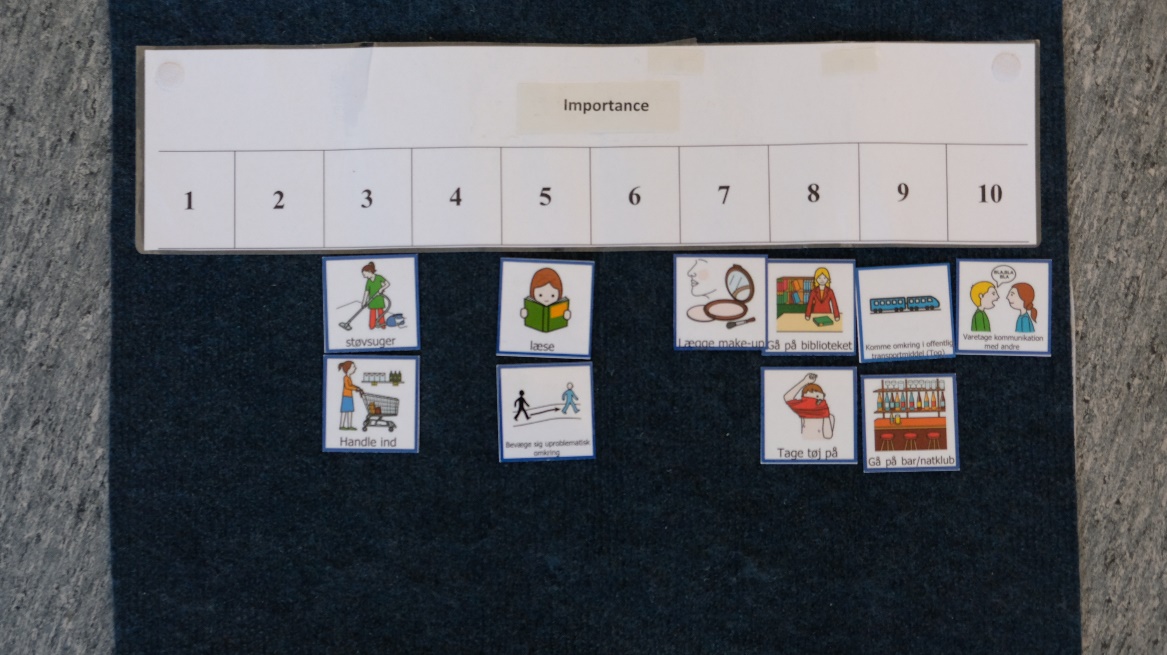


**Step 3: Scoring performance of occupations**


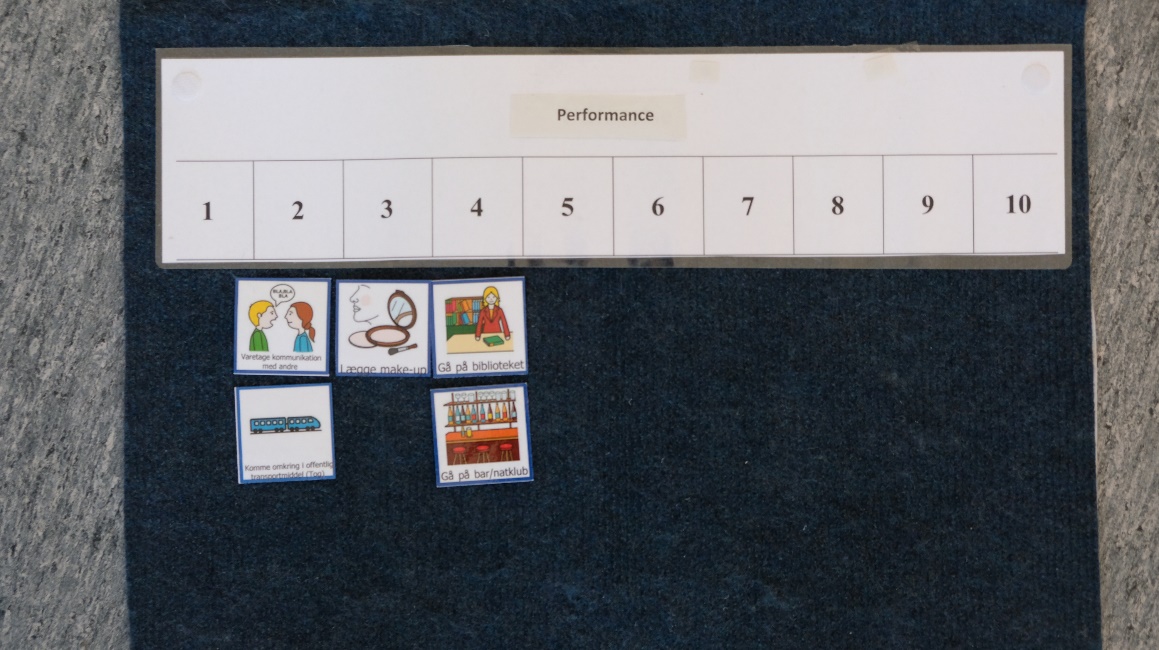


**Step 4: Scoring satisfaction**


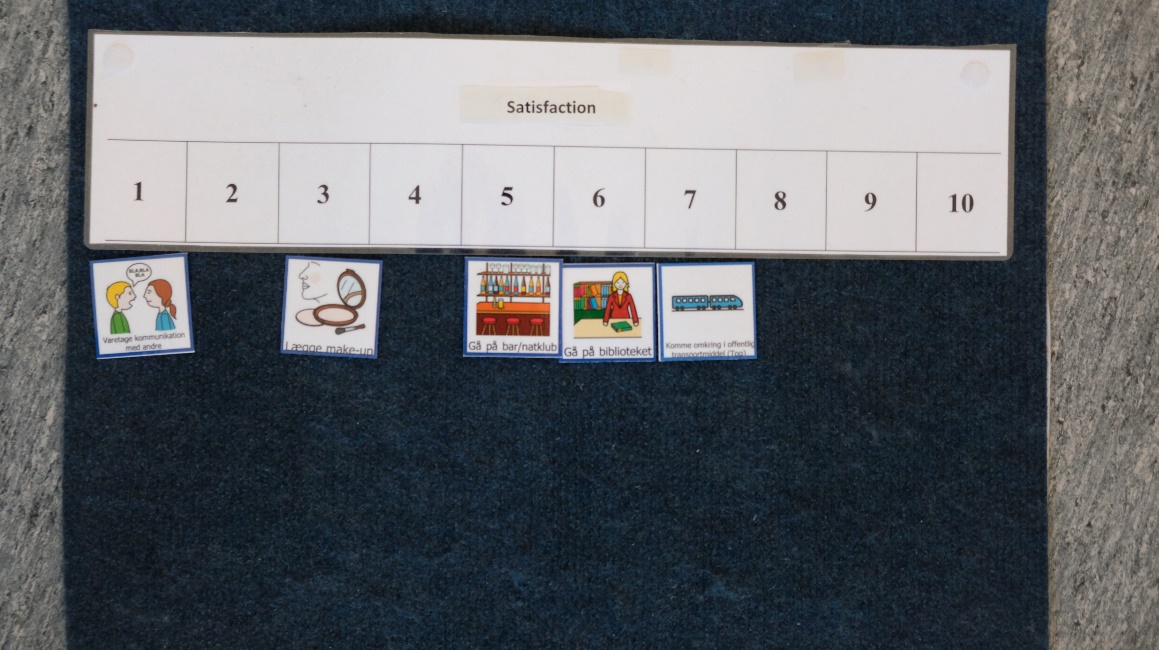

Supplement: Supplementary Materials — Appendix 1: “questionnaire covering three areas related to the level of client-centeredness” shows the questionnaire that the evaluation team has used to score the compliance and the quality of the COPM interview. Appendix 2: “example of a completed COPM interview supported by Talking Mats.” The example includes occupational performance within self-care, productivity, and leisure. [file 9367315.f1.doc]
